# A novel hand-eye calibration method of picking robot based on TOF camera

**DOI:** 10.3389/fpls.2022.1099033

**Published:** 2023-01-17

**Authors:** Xiangsheng Zhang, Meng Yao, Qi Cheng, Gunan Liang, Feng Fan

**Affiliations:** ^1^ Key Laboratory of Advanced Process Control for Light Industry, Ministry of Education, Jiangnan University, Wuxi, Jiangsu, China; ^2^ Visual Algorithm R&D Department, XINJIE Electronic Limited Company, Wuxi, Jiangsu, China

**Keywords:** fruit picking, picking robot, hand-eye calibration, TOF camera, point cloud

## Abstract

Aiming at the stability of hand-eye calibration in fruit picking scene, a simple hand-eye calibration method for picking robot based on optimization combined with TOF (Time of Flight) camera is proposed. This method needs to fix the TOF depth camera at actual and calculated coordinates of the peach the end of the robot, operate the robot to take pictures of the calibration board from different poses, and record the current photographing poses to ensure that each group of pictures is clear and complete, so as to use the TOF depth camera to image the calibration board. Obtain multiple sets of calibration board depth maps and corresponding point cloud data, that is, “eye” data. Through the circle center extraction and positioning algorithm, the circle center points on each group of calibration plates are extracted, and a circle center sorting method based on the vector angle and the center of mass coordinates is designed to solve the circle center caused by factors such as mirror distortion, uneven illumination and different photographing poses. And through the tool center point of the actuator, the coordinate value of the circle center point on the four corners of each group of calibration plates in the robot end coordinate system is located in turn, and the “hand” data is obtained. Combined with the SVD method, And according to the obtained point residuals, the weight coefficients of the marker points are redistributed, and the hand-eye parameters are iteratively optimized, which improves the accuracy and stability of the hand-eye calibration. the method proposed in this paper has a better ability to locate the gross error under the environment of large gross errors. In order to verify the feasibility of the hand-eye calibration method, the indoor picking experiment was simulated, and the peaches were identified and positioned by combining deep learning and 3D vision to verify the proposed hand-eye calibration method. The JAKA six-axis robot and TuYang depth camera are used to build the experimental platform. The experimental results show that the method is simple to operate, has good stability, and the calibration plate is easy to manufacture and low in cost. work accuracy requirements.

## 1 Introduction

In recent years, the output of agricultural products in my country has increased year by year. By 2022, China’s output of fruits and vegetables will rank first in the world. At present, fruit and vegetable picking is mainly based on manual picking. However, with the process of urbanization, the shortage of labor has been exacerbated, resulting in a substantial increase in the cost of fruit and vegetable picking. Therefore, the development of picking robots that can improve production efficiency and reduce picking costs is an inevitable trend in fruit and vegetable production.

Since the 1980s, various systematic theoretical researches on picking robots have been carried out at home and abroad (Willigenburg et al., 2004; Ji et al., 2011; Gan et al., 2018; Li and Tian, 2018; Tian et al., 2019). The working environment of picking robots is complex, there are many uncertain factors, and picking is difficult. An efficient, fast and stable fruit and vegetable picking robot system is urgently needed. And picking robotics robots are enabled by technologies not available in the earlier literature era, including huge advances in computing power and speed, advanced machine vision and imaging systems, image processing techniques, artificial intelligence techniques, and improved gripping and handling. As a result, in 2020-2021, picking robots will face a highly competitive market, and many large companies will introduce new reliable and efficient picking robots (Bogue, 2020). Studies have shown that the correlation and precise positioning of vision and robots, that is, hand-eye calibration, are the key technologies and prerequisites for realizing active vision and automatic picking of picking robots (Tian et al., 2019). The main purpose of hand-eye calibration is to obtain the conversion relationship from the camera to the end of the robot, which is convenient for controlling the robot’s arm to complete the corresponding task. Therefore, the research on hand-eye calibration is of great significance to the picking robot.

There are two ways to associate vision and robots: Eye-to-Hand and Eye-in-Hand. In the process of researching picking robots, it is found that active vision measurement has better advantages in outdoor picking, because compared with the situation where the camera is fixed in the Eye-to-Hand association method, the camera is bound to the end of the robotic arm. As the robotic arm changes the shooting pose, it can overcome the influence of uncertain factors such as uneven ground, wind disturbance, and illumination changes in the wild environment (Mo et al., 2017). The Hand-eye system is the main way for picking robots to achieve active vision (Xu et al., 2008).

As early as the 1980s, Tsai and Daniilidis et al. (Tsai and Lenz, 1989; Chen, 1991; Daniilidis, 1999; Malm and Heyden, 2000) proposed a classic hand-eye calibration method for the Eye-in-Hand vision and robot correlation method, which are mainly divided into “two-step method” and “one-step method”, and its mathematical model boils down to solving the AX=XB matrix equation. After that, various more efficient hand-eye calibration algorithms and mathematical models have been proposed one after another (Malti, 2013; Nobre and Heckman, 2019; Koide and Menegatti, 2019; Deng et al., 2021; Fei et al., 2021; Liu et al., 2021; Pedrosa et al., 2021; Wang and Min, 2021; Wu et al., 2021). In China, there have also been some researches on the hand-eye calibration method of picking robots. For example, Mo Yuda of South China Agricultural University (Mo et al., 2017) proposed a hand-eye calibration optimization method to solve the homogeneous transformation matrix equation and applied it to the litchi picking robot. “Two-step method” solves the problem of error transmission to optimize results; and Jin Yucheng (Jin et al., 2021) proposed a deep vision hand-eye coordination planning strategy to solve the problems of low picking accuracy and efficiency of existing picking robots. Abroad, University of Wageningen developed a new hand-eye sensing and servo control framework and applied it to picking robots in dense vegetation (Barth et al., 2016).

In recent years, with the development of depth cameras, depth cameras have been increasingly applied to the field of visual picking (Wang et al., 2018; Zhang et al., 2021; Pan and Ahamed, 2022), which has improved the recognition ability and efficiency of fruit and vegetable picking. In addition, depth cameras have also been applied. In hand-eye calibration (Ðurović et al., 2017; Du et al., 2018; Ge et al., 2022). TOF (Time of flight) depth camera is a camera with active vision measurement function. Its working principle is to continuously send light pulses to the target, and then use the sensor to receive the light returned from the object, by detecting the flight round-trip time of the light pulses Compared with other 3D cameras, it has the advantages of cheap price, small size, low power consumption, strong anti-light interference and fast calculation of depth information (Li et al., 2021), which is very suitable for the application of wild fruit picking.

This paper studies a hand-eye calibration method combined with a depth camera. It adopts the eye-in-hand correlation method, combines the TOF depth camera to take pictures of fixed feature points, and uses the TCP contact measurement method to obtain the hand-eye data, which is transformed into the solution of the equation AX=B, and then iteratively optimizes the hand-eye parameters by changing the weight coefficient of the marker points to obtain the final hand-eye calibration matrix, and the hand-eye calibration method is verified in the indoor simulated peach picking experiment. The main work is as follows:

1) The traditional circular calibration plate used for picking is modified and designed to facilitate the contour extraction and center positioning of the circular target, as well as the tool center point (TCP) of the auxiliary robot end effector to accurately measure the center point base mark.
2) A circle center sorting method based on the vector angle and the center of mass coordinates of the circular calibration plate is proposed, which can solve the problem of out-of-order extraction of circle centers and specify the sorting direction.
3) Combined with the SVD algorithm, the hand-eye matrix is solved, the residuals of the points are estimated, the weight coefficients of the marker points are redistributed, and the hand-eye parameters are iteratively optimized, which improves the accuracy and stability of the hand-eye calibration of the picking robot.
4) A method for peach identification and localization is designed, combined with the deep network model and TOF camera, to verify the reliability of the hand-eye calibration method.

## 2 Hand-eye calibration scheme of picking robot

### 2.1 Hand-eye calibration model

**Figure 1:**
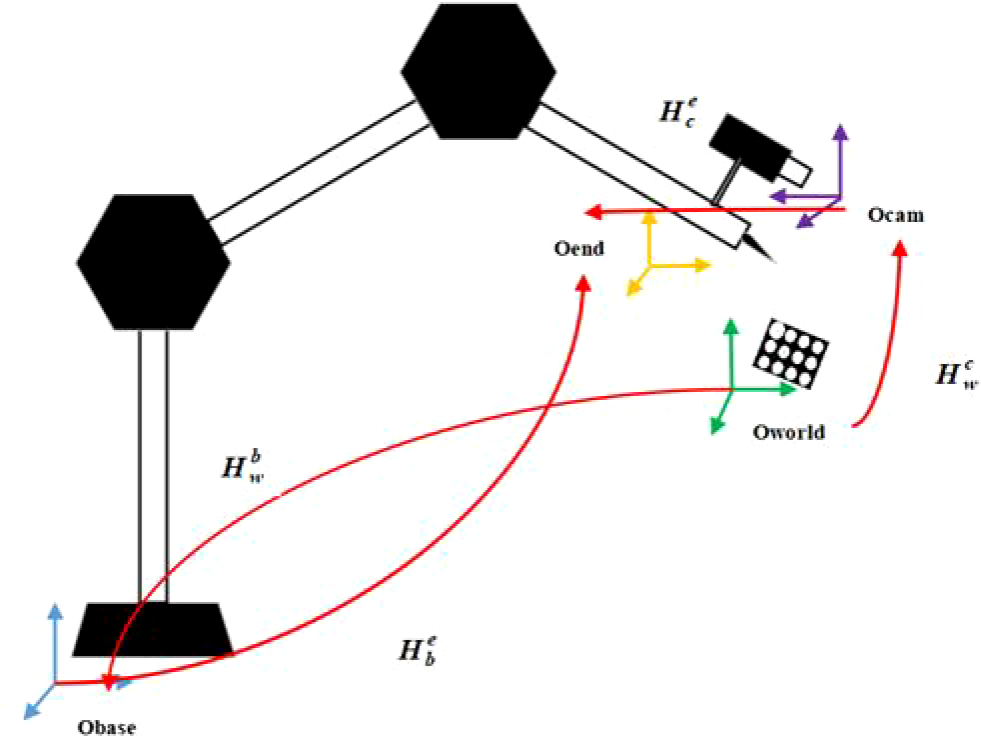
Hand-eye calibration model of picking robot.

This method mainly studies the hand-eye calibration model of the eye on the hand, that is, the camera is fixed at the end of the robotic arm. As shown in **Figure 1**, *O_base_* represents the base coordinate system of the robot, *O_end_* represents the execution end coordinate system of the robot, a camera is installed at the end of the robot, *O_cam_* is the camera coordinate system, and a calibration board is fixed in the robot’s field of view, and *O_world_* is the world coordinate system. (Calibration board coordinate system), these four coordinate systems and their mutual conversion relationship constitute the mathematical model of the picking robot’s hand-eye vision system. The relationship between the calibration board and the robot base coordinates is fixed. Hand-eye calibration.

The TOF camera can realize the conversion from the world coordinate system *O_world_* to the camera coordinate system *O_cam_*, and the conversion relationship is $H_{w}^{c}$ ; $H_{w}^{b}$ . Indicates the conversion from the world coordinate system *O_world_* to the robot base coordinate system *O_base_*, and the position of the calibration board in the base coordinate system can be obtained; the motion feedback data of the robot can obtain through the conversion relationship $H_{b}^{e}$ from the robot base coordinate system *O_base_* to the execution end coordinate system *O_end_*; $H_{c}^{e}$ represents the conversion relationship from the camera coordinate system *O_cam_* to the execution end coordinate system *O_end_*. The above four transformation relationship matrices are the key information to realize the positioning and guidance of the robot.

### 2.2 Design of hand-eye calibration method

First, TCP calibration is carried out for the robot, and the ‘Four point calibration method (Liu et al., 2012)’ is used to calibrate the position of TCP in the end coordinate system of the robot. Then, the end of the robot is operated to locate the fixed mark points on the calibration board by contact, and the TOF depth camera is used to take pictures of the calibration board from different poses. The calibration method is as follows:

1) First, teach the robot’s pose to ensure that the camera can capture a complete and clear picture of the calibration board in each group of poses. Then, the host computer sends the “start calibration” command, and the robot takes pictures of the calibration board according to the taught pose, and can obtain *m*(*m*∈[5,10]) group depth pictures, point cloud information, and pose coordinates when the robot takes pictures.
2) Control the end effector (needle tip) of the robot, in the fixed order of upper left, upper right, lower left and lower right, respectively contact the center points on the four corners of the calibration plate to obtain a set of homogeneous coordinates of the center points under the robot base coordinate system *O_base_*;

$$P_{Basei}=\left[\begin{array}{ccc} x_{Bi} & y_{Bi} & \begin{array}{cc} z_{Bi} & 1 \end{array} \end{array}\right](i=0,1,2,3)$$

3) After processing the depth images of each calibration plate collected by the TOF camera, $P_{Cami}=\left[\begin{array}{ccc} x_{Ci} & y_{Ci} & \begin{array}{cc} z_{Ci} & 1 \end{array} \end{array}\right](i=0,1,2,3)$ :a set of three-dimensional center point homogeneous coordinates can be obtained by combining the point cloud coordinates and the index numbers of the circle center points on the four corners of the calibration plate through the circle center positioning algorithm, and get the pose parameters of the robot *P_T_* at this time from the robot controller, *x_T_* 、*y_T_* 、*z_T_* is the position vector of the end of the robot tool end coordinate system in the robot base coordinate system, and *R*_*x*_*T*__ 、 *R*_*y*_*T*__ 、 *R*_*z*_*T*__ is the Euler angle of each joint of the robot under the robot posture at this time;

$$P_{T}=\left[\begin{array}{ccc} x_{T} & y_{T} & \begin{array}{cc} z_{T} & \begin{array}{cc} Rx_{T} & \begin{array}{cc} Ry_{T} & Rz_{T} \end{array} \end{array} \end{array} \end{array}\right]$$

4) Through each set of pose coordinates, obtain the three-dimensional homogeneous coordinates of the circle center point under the robot base coordinate system *O_base_* under the execution end coordinate system *O_end_*, which is obtained by formula (1):

(1) $$P_{End}=H_{b}^{e}*P_{Base}$$

Among this, $P_{End}=\left[\begin{array}{cccc} x_{E} & y_{E} & z_{E} & 1 \end{array}\right]$ , $P_{Base}=\left[\begin{array}{cccc} x_{B} & y_{B} & z_{B} & 1 \end{array}\right]F$ ,decompose $H_{b}^{e}$ into rotation part $R_{b}^{e}$ (3x3) and translation part $T_{b}^{e}$ (3x1), $R_{b}^{e}$ can be got by formula (2):


(2) $$R_{b}^{e}=R_{x}*R_{y}*R_{z}$$

in formula(2), *R_x_*, *R_y_* and *R_z_* are respectively:


$$R_{x}=\left[\begin{array}{ccc} 1 & 0 & 0\\ 0 & \cos(Rx_{T}) & -\sin(Rx_{T})\\ 0 & \sin(Rx_{T}) & \cos(Rx_{T}) \end{array}\right]$$

$$R_{\text{y}}=\left[\begin{array}{ccc} \cos(Ry_{T}) & 0 & \sin(Ry_{T})\\ 0 & 1 & 0\\ -\sin(Ry_{T}) & 0 & \cos(Ry_{T}) \end{array}\right]$$

$$R_{z}=\left[\begin{array}{ccc} \cos(Rz_{T}) & -\sin(Rz_{T}) & 0\\ \sin(Rz_{T}) & \cos(Rz_{T}) & 0\\ 0 & 0 & 1 \end{array}\right]$$

$T_{b}^{e}$ is obtained by formula(3):


(3) $$T_{b}^{e}=\left[\begin{array}{c} x_{T}\\ y_{T}\\ z_{T} \end{array}\right]$$

5) Extract the homogeneous coordinates of the center of the point cloud corresponding to each set of calibration plates under the camera coordinate system *O_cam_ : P_CamAll_*, and the homogeneous coordinates of all circle centers under the end coordinate system *O_end_* obtained by formula(1): *P_EndAll_*, *n*=4*m,

$$P_{CamAll}=\left[\begin{array}{ccc} \begin{array}{c} x_{C0}\\ x_{C1}\\ \vdots \\ x_{Cn} \end{array} & \begin{array}{c} y_{C0}\\ y_{C1}\\ \vdots \\ y_{Cn} \end{array} & \begin{array}{c} z_{C0}\\ z_{C1}\\ \vdots \\ z_{Cn} \end{array} \end{array}\right]$$

$$P_{EndAll}=\left[\begin{array}{ccc} \begin{array}{c} x_{E0}\\ x_{E1}\\ \vdots \\ x_{En} \end{array} & \begin{array}{c} y_{E0}\\ y_{E1}\\ \vdots \\ y_{En} \end{array} & \begin{array}{c} z_{E0}\\ z_{E1}\\ \vdots \\ z_{En} \end{array} \end{array}\right]$$

thus, the conversion relationship from the camera coordinate system *O_cam_* to the execution end coordinate system *O_end_*: $H_{c}^{e}$ is obtained, then $H_{c}^{e}$ can be decomposed into rotation matrix $R_{c}^{e}$ (3x3) and translation vector $T_{c}^{e}$ (3x1),as shown in formula(4):


(4) $$P_{EndAll}=R_{c}^{e}*P_{CamAll}+T_{c}^{e}$$

## 3 Hand-eye calibration algorithm and optimization

### 3.1 Calibration plate design

Compared with the traditional round calibration board, the calibration board designed in this paper is simple to make and has lower cost. One A4 paper is enough, and it does not need to ensure high-precision size spacing. It can also assist TCP to locate the center of the circle and ensure the consistency of the order of extracting the center of the circle. The improvements are as follows:

1) Ensure that the circular target in the upper left corner is significantly larger than other circular targets in any photographing posture, which is helpful for the subsequent sorting of marker points;
2) The circular target is required to be separated from the background, and the colors should be very different, so that the outline of the circular target can be quickly extracted;
3) The size of the calibration plate should be such that when the camera is shooting at close range, the camera field of view can cover the entire calibration plate;
4) In the center of each circular target, draw a “ × “ symbol, which is helpful for the precise positioning of the contact center by the end of the execution.

**Figure 2:**
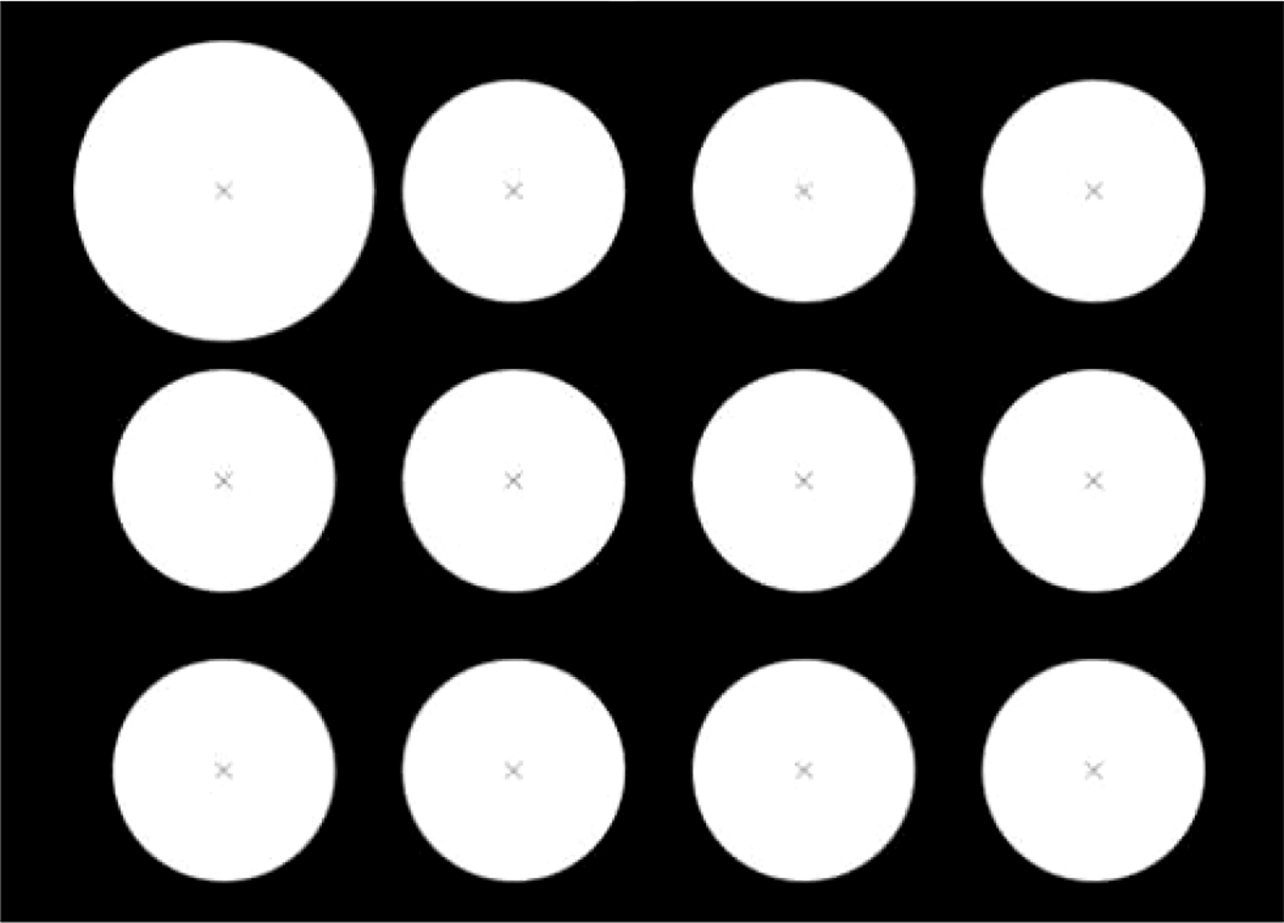
Designed calibration board.

The designed calibration board is shown in **Figure 2**.

### 3.2 Algorithm for center positioning and sorting

The circular calibration plate generally takes the center of the characteristic circle as the feature point, adopts the center detection and positioning algorithm, first preprocesses the image of the calibration plate, then performs Canny edge detection, and then sets the conditions such as roundness and area to screen the obtained contour, and finally Use least squares ellipse fitting to obtain the center of the circle. The method has low real-time performance, but has strong robustness and high precision to the influence of external factors, and can reach the sub-pixel level.

**Figure 3:**
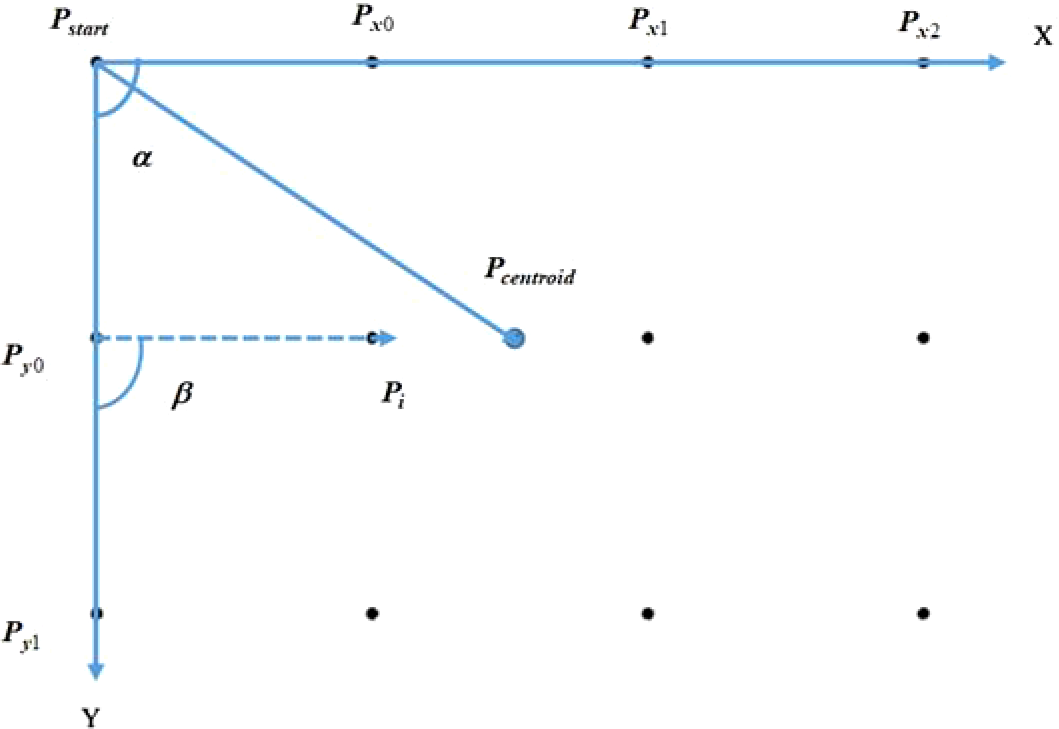
Schematic diagram of center sorting.

**Figure 4:**
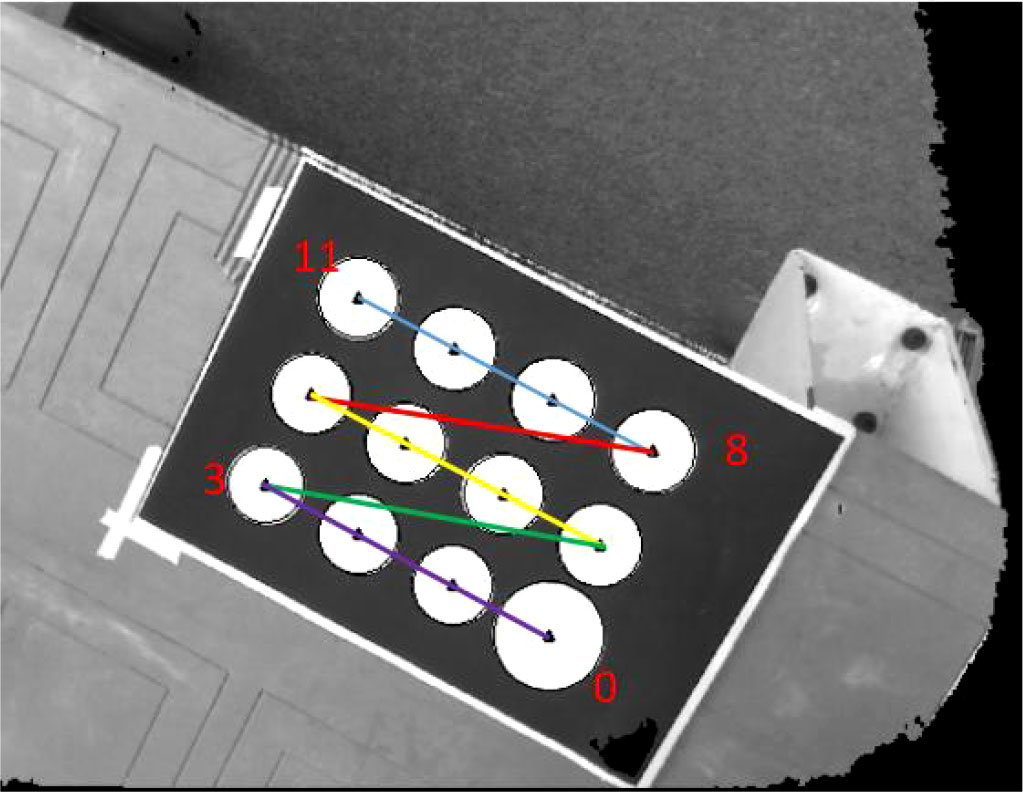
Schematic diagram after sorting the center.

Aiming at the problem of out-of-order extraction of center points caused by factors such as mirror distortion, uneven illumination and different photographing poses, a center sorting method based on vector angles and centroid coordinates is adopted to make the center lattice sorting algorithm with rotation invariance. The coordinates of the center of the circle are extracted in the order of X direction and then Y direction. The schematic diagram of the sorting of circle points is shown in **Figure 3**. The method is as follows:

1) Determine the initial point, according to the circular contour obtained by Canny edge detection and contour screening, search the contour with the most subpixel points on the contour, and then carry out least squares ellipse fitting to obtain the initial point *P_start_*;
2) Calculate the Euclidean distances from other circle centers to the initial point respectively, and sort them from small to large according to the Euclidean distance, and select the two nearest points, namely *P_x 0_* and *P_y0_*;
3) Find the centroid coordinates of all the circle centers *P_centroid_*, and get three direction vectors, $\vec{P_{start}P_{centroid}}$ , $\vec{P_{start}P_{x0}}$ ,and $\vec{P_{start}P_{y0}}$ , with the starting point is the *P_start_*. The formula (5) is introduced below to judge the positional relationship between the vectors:

(5) $$\vec{A}=(x_{1},y_{1})\;\vec{B}\text{=(}x_{2},y_{2}\text{) }\vec{A}⊗\vec{B}=x_{1}y_{2}-x_{2}y_{1}$$

if $\vec{A}⊗\vec{B}>0$ , $\vec{B}$ is in the counterclockwise direction of $\vec{A}$ ; if $\vec{A}⊗\vec{B}<0$ , $\vec{A}$ is in the clockwise direction of $\vec{B}$ ; if $\vec{A}⊗\vec{B}$ =0, $\vec{B}$ is collinear with $\vec{A}$ ; from this, it can be judged that the positional relationship of the three direction vectors $\vec{P_{start}P_{centroid}}$ , $\vec{P_{start}P_{x0}}$ ,and $\vec{P_{start}P_{y0}}$ . In this article, let $\vec{P_{start}P_{x0}}$ is in the counterclockwise direction of $\vec{P_{start}P_{centroid}}$ , that is, represent the *X* direction; $\vec{P_{start}P_{y0}}$ is in the clockwise direction of $\vec{P_{start}P_{centroid}}$ , that is, represent *Y* direction;

4) Calculate the angle α between the vector from the center of the other circle to the starting point and the *X* direction, set the angle threshold, the reference point in the *Y* direction is the one that meets the conditions, and sort according to the Euclidean distance from the initial point from small to large, you can get and;
5) Take the reference point in the *Y* direction as the reference point in turn, calculate the angle β between the vectors from the center of the other circles to the reference point and the Y direction, set the angle threshold, filter out the center of the circle that is collinear with the *X*direction, and follow the distance with the reference point. Euclidean distance is sorted from smallest to largest. The sequence diagram of the center of the circle after sorting is shown in **Figure 4**.

After the sorting is completed, a set of sub-pixel coordinates is obtained. It is necessary to precisely locate the three-dimensional coordinates of the marker point in the point cloud coordinate system. Assuming that the sub-pixel coordinates of the extracted marker point is *P=(u,v*), then the coordinates of the four surrounding integer pixels are *P_0_* =(*u_0_, v_0_*), *P_1_* =(*u*_0_+1,*v*_0_), *P_2_* =(*u*_0_,*v*_0_+1), P_3_=(*u_0_* +1, *v_0_* +1), Then, a method proposed by Cheng Qi (Cheng et al., 2021) using the reciprocal of the subpixel area ratio as the weight of spatial point interpolation is used to accurately estimate the point cloud coordinates of the marker points.

### 3.3 Solving the initial hand-eye calibration matrix

The hand-eye calibration method used in this paper is to convert the data under the camera coordinate system *O_cam_* to the execution end coordinate system *O_end_*. This process can be regarded as the rigid body transformation of the point cloud, which can be solved by the method of SVD and least squares. The calculation accuracy and efficiency is high, its mathematical model is:


(6) $$F(R,T)=\arg\min\sum_{i=1}^{n}w_{i}||R\cdot P_{CamAlli}+T-P_{EndAlli}||$$

in the formula, R is the rotation matrix, T is the translation matrix,*w_i_* is the weight coefficient, and the initial value is set to 1; *n* is the number of marker points. By taking the partial derivative of formula (10), the minimum value can be obtained, which can be simplified and sorted as follows:


(7) $$R\cdot {\bar{P}}_{CamAll}+T={\bar{P}}_{EndAll}$$

where ${\bar{P}}_{CamAll}$ and ${\bar{P}}_{EndAll}$ are respectively the centroid points of the set of points, defined as:


(8) $${\bar{P}}_{CamAll}=(\sum_{i=1}^{n}w_{i}P_{CamAll})/\sum_{i=1}^{n}w_{i}$$

(9) $${\bar{P}}_{EndAll}=(\sum_{i=1}^{n}w_{i}P_{EndAll})/\sum_{i=1}^{n}w_{i}$$

Substitute equation (7) into (6) and eliminate T, then can get


(10) $$F(R,T)=\arg\min\sum_{i=1}^{n}w_{i}||R\cdot P'CamAlli-P^{'}EndAlli||$$

where *P* ^'^__*CamAlli*__ = *P*_*CamAll*_ - ${\bar{P}}_{CamAll}$ 、 *P* ^'^_*EndAlli*_ = *P*_*EndAll*_ - ${\bar{P}}_{\mathrm{EndAll}}$ , the optimization problem can be transformed into:


(11) $$R=\underset{R}{\arg\min}\frac{1}{2}\sum_{i=1}^{n}w_{i}||R\cdot P^{'}CamAlli-P^{'}EndAlli||$$

convert equation (11) into:


(12) $$R=\arg\max(\sum_{i=1}^{n}w_{i}P^{'}EndAlliTRP^{'}CamAlli)$$

Let $H=\sum_{i=1}^{n}P'CamAlliwiP^{'}{EndAlli}_{T}$ , and perform SVD decomposition on H:


(13) $$H=U\Lambda V^{T}$$

Where *U* is a left singular matrix, *V* is a right singular matrix, and *Λ* is a diagonal matrix.

Finally get:


(14) $$R=\arg\max(tr(RU\Lambda V^{T}))$$

In the formula,*U*, *V*, *Λ* are all orthogonal matrices, at this time:


(15) $$R=VU^{T}$$

(16) $$T={\bar{P}}_{EndAll}-R\cdot {\bar{P}}_{CamAll}$$

### 3.4 Weighted iterative method to optimize hand-eye calibration parameters

During the calibration process, the camera itself is easily affected by the environment (light, temperature, humidity, pressure, etc.), which affects the measurement accuracy; at the same time, some human factors will also cause large errors in the measurement, such as the end of the operation robot contacting and positioning the center of the circle. If it is large, a large gross error is introduced into the feature points. At this time, if the calculation method of equal weight is used for coordinate system transformation, the stability of the SVD algorithm will be reduced. Therefore, the allocation of weight coefficients needs to be considered during the execution of the algorithm.

The point residual for each marker point is:


(17) $$e_{i}=P_{EndAlli}-(RP_{CamAlli}+T)$$

At this time, the absolute mean value of the point residuals is:


(18) $$\bar{e}=\sum_{i=1}^{n}e_{i}$$

The weight coefficients are redistributed according to the residual error of each marker point, so that the weight of the measurement point with larger error is reduced, thereby increasing the stability of the SVD algorithm. Here, take the weight function as the Danish (Wang, 2006) function:


(19) $$w_{i}=\{\begin{array}{c} 1\begin{array}{c} \begin{array}{c} \begin{array}{c} \left|u_{i}\leq k_{0}\right| \end{array} \end{array} \end{array}\\ \exp\left[1-{\left(u_{i}/k_{0}\right)}^{2}\right]\begin{array}{c} \left|u_{i}>k_{0}\right| \end{array} \end{array}$$

In the formula, *u*_*i*_=*e*_*i*_/*σ* , *σ* is the error in the point position, $\sigma =\sqrt{\sum_{i=1}^{n}e_{i}^{2}/n}$ , *k*_0_ is the harmonic coefficient of the weight function, usually 1.0~2.5, this paper takes *k*_0 =_ 2.5.The weight coefficient matrix corrected according to equation (19) is re-substituted into equation (6) for iterative solution. If the number of iterations or the error threshold is reached, the iteration is terminated.

## 4 Experimental results and analysis

### 4.1 Experimental equipment

**Figure 5:**
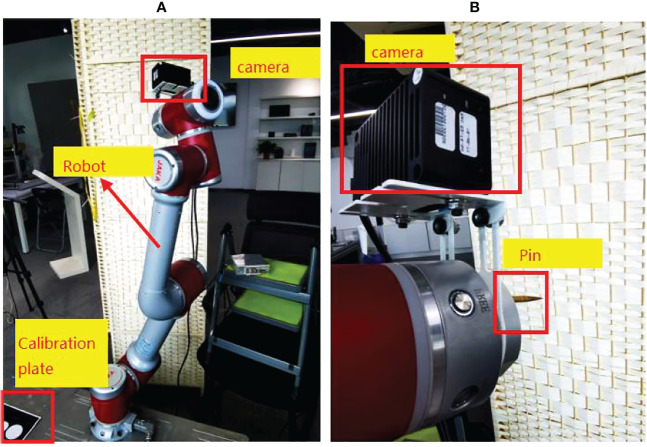
**(A)** Experimental equipment Experimental platform **(B)** End effector and camera.

The built experimental platform is shown in **Figure 5**. The image acquisition device is TuYang TM460-E2 TOF camera, the working distance is 0.1m-2.4m, the size is 87.4mm*51.5mm*38mm, the weight is 257g, and the depth image resolution is 640 *480, this depth camera is small in size and light in weight, which is very suitable for outdoor picking; the picking robot is JAKA Zu 5, the end load is 5kg, and the repeat positioning accuracy is ±0.02mm; the vision software is independently developed, and the development environment Visual Studio, The development language is C++.

### 4.2 Experiment results and analysis of hand-eye calibration

In order to test the effect and robustness of the weighted SVD method proposed in this paper, a comparison experiment will be carried out under two different conditions of no gross error and gross error. The comparison experiments include SVD algorithm, LM algorithm, Danish-DQ (Cheng et al., 2021),and the Danish-SVD proposed in this paper. In the case of no noise and no gross error, the average absolute error of the point is shown in **Table 1**:

**Table 1:** $\bar{e}$ of each weight function without gross error.

Evaluation indicators	SVD		LM	Danish-DQ	Danish-SVD
$\bar{e}$ /mm	4.18036		4.00379	4.00294	3.89256

It can be seen from **Table 1** that the LM algorithm, the Danish-DQ algorithm and the Danish-SVD algorithm proposed in this paper can improve the accuracy of hand-eye calibration, but compared to the algorithm proposed in this paper, the effect is better.

The Danish weight function used in this paper is to reduce the influence of gross error by re weighting the residual error. This paper will conduct comparative experiments with other common weight functions, such as Huber function, IGG I function, IGG III function, to verify the advantages of Danish weight function. The experimental results are shown in **Table 2**:

**Table 2:** $\bar{e}$ of each algorithm without gross error.

Evaluation indicators	Danish	Huber	IGG I	IGG III
$\bar{e}$ /mm	3.89256	3.97189	3.92653	3.91756
Iterations	7	10	8	9

As can be seen from **Table 2**, the Danish weight function used in this paper is better than other weight functions in terms of precision and iteration number. However, the overall average absolute error value is relatively large. The reason is analyzed from the absolute error of each marker point in the X, Y, and Z axis directions under the operation result of the Danish-SVD algorithm proposed in this paper.

**Figure 6:**
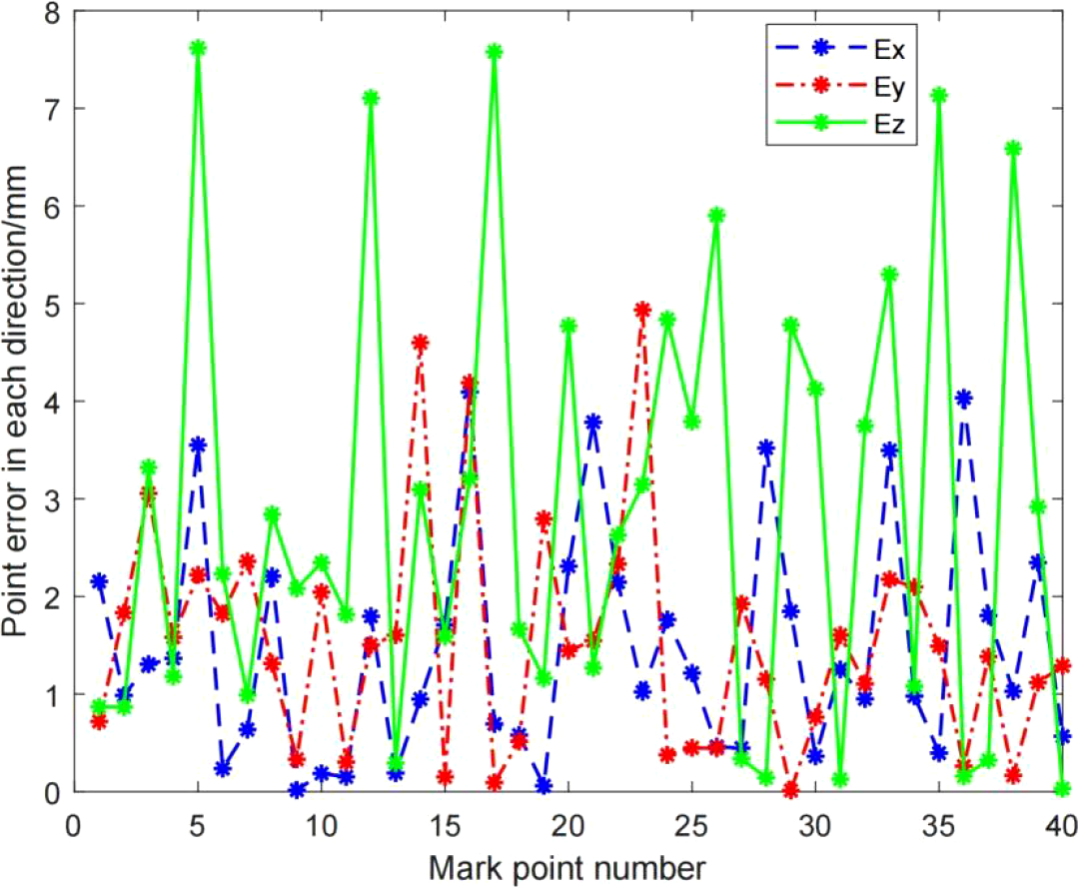
The absolute error of the mark point in the X, Y, Z axis directions.

As can be seen from **Figure 6** and **Table 3**, the absolute error of the marker point in the Z-axis direction is larger, and the accuracy of this TM460-E2 depth camera in the Z-direction is 5mm-20mm, combined with the working principle of the TOF camera, and after analyzing the data in **Table 4**, it is concluded that the higher the camera pose during hand-eye calibration, the greater the absolute error. Therefore, when teaching the camera pose, it is required that the camera can shoot a complete circular calibration plate. pose as low as possible. And the resolution of this TOF camera is only 640*480. The lower resolution is also the main reason for the large mean absolute error.

**Table 3:** The maximum value, minimum value and average value of the absolute error of the mark point in the X, Y, Z axis directions.

Evaluation indicators	*E_x_*	*E_y_*	*E_z_*
maximum/mm	4.02153	4.73257	7.45387
minimum/mm	0.015798	0.0118846	0.0323323
average/mm	1.365	1.4276	2.67459

**Table 4:** The maximum value, minimum value and average value of the absolute error of the marker points in the Z-axis direction and the value of $\bar{e}\;$ at different heights.

Evaluation indicators	far	close
maximum of *E_z_*/mm	12.6868	7.61753
minimum of *E_z_*/mm	0.06296	0.0323323
Average of *E_z_*/mm	4.84461	2.87459
$\bar{e}$ /mm	6.52849	3.95187

**Figure 7:**
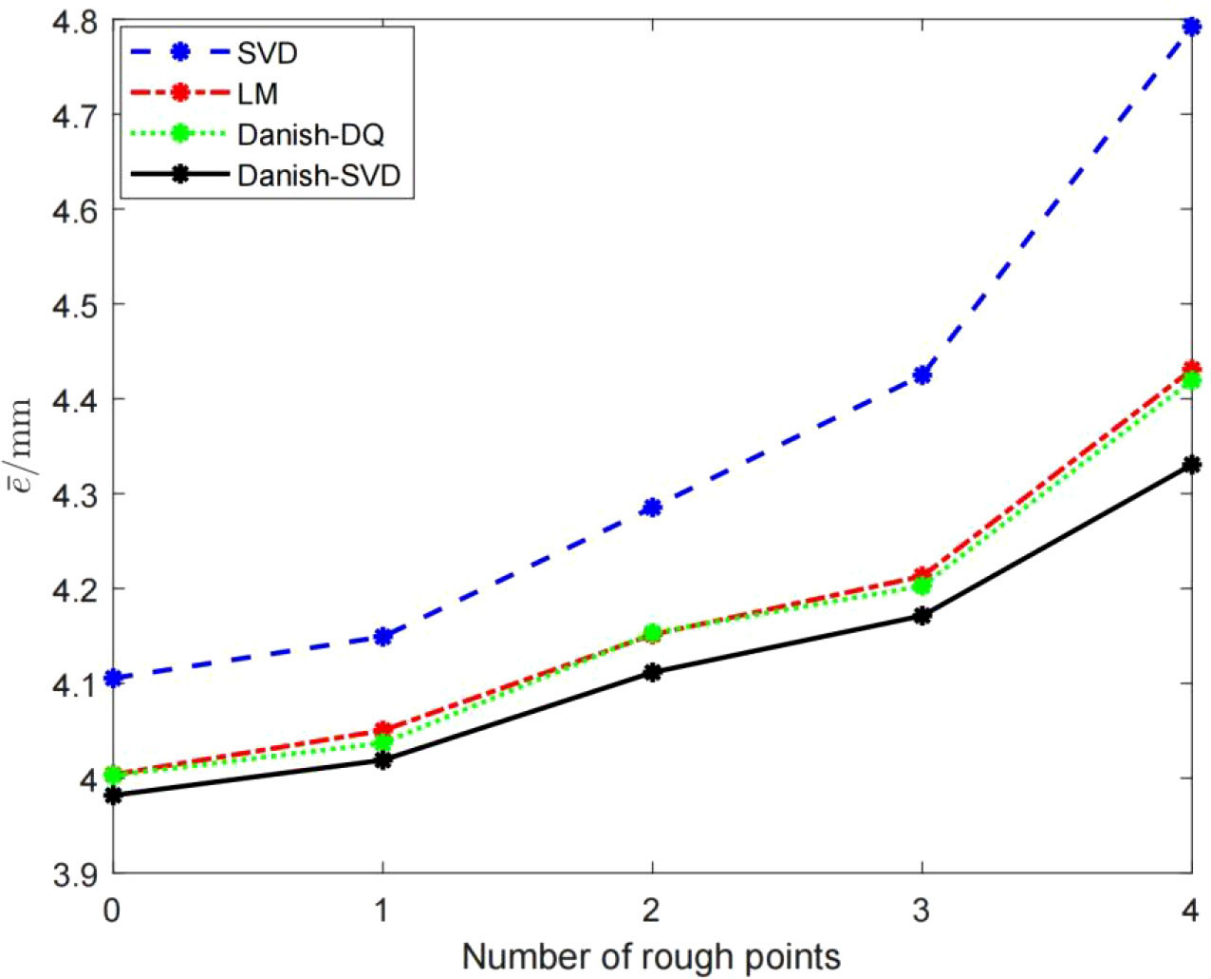
Each $\bar{e}\;$ of algorithm in the case of introducing gross error.

Considering the robustness requirements of outdoor picking for field hand-eye calibration, this paper adds (1, 0, 1), (-1, 0, 1),(0,-1,0), (1,-1,0), which the gross error in mm to the marker points in the order of contact and positioning marker points in the point set *P_Base_*, simulates the gross error introduced by human factors caused by the mis-operation of contact positioning, each algorithm has different numbers of gross error points The absolute error of the point below is shown in **Figure 7**. It can be seen that the LM algorithm, the Danish-DQ algorithm and the Danish-SVD algorithm proposed in this paper have better resistance to errors than the SVD method, but the performance of the method proposed in this paper is better than that of the SVD method.

**Figure 8:**
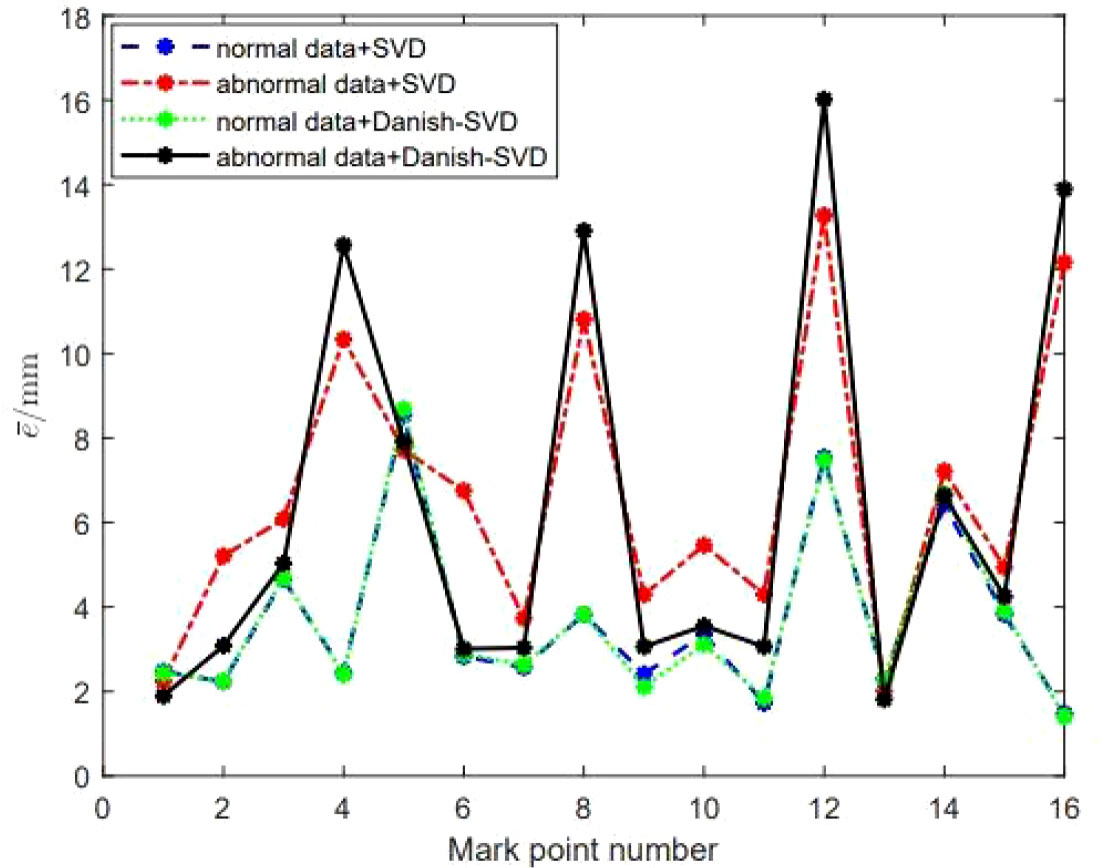
Performance comparison of SVD and Danish-SVD algorithms under the condition of introducing large gross errors.

In order to test the ability of the Danish-SVD method proposed in this paper to locate the gross error, the mark point with the serial number of 3 in the point set *P_Base_* refers to (10, 10, 10), which the gross error in mm, and the mark points of the first four groups of calibration plates are taken. After analyzing the data, it can be seen from **Figure 8** that the absolute errors of the other three markers are closer to the SVD method under normal data and interference data, and can more effectively locate the position of gross errors, indicating that the Danish-SVD algorithm proposed in this paper is used. It can more effectively suppress the influence of a single interference data, and has a strong ability to identify gross errors.

### 4.3 Experiment results and analysis of peach picking

The peach picking experiment is simulated in an indoor environment, and the reliability of the hand-eye calibration method proposed in this paper is verified according to the positioning accuracy of peach picking. The experimental design is as follows:

1) Collect peach pictures, use the MVTEC DLT tool to label the pictures, call the HALCON deep network model to train the pictures, and obtain the training model;
2) Perform hand-eye calibration on the robot to obtain the conversion relationship from the camera coordinate system *O_cam_* to the robot execution end coordinate system *O_end_*;
3) Shoot the peaches to be picked (9.a, 9.b), perform correction and registration to obtain a color point cloud (9.c), call the training model to process the color image of the peaches, and identify and locate the peaches (9.d), map the positioning coordinates to the point cloud, and segment the peach point cloud (9.e);
4) Using the hand-eye conversion relationship, convert the coordinates of the peach point cloud to the execution end coordinate system *O_end_*, and obtain the coordinates of the center of mass, that is, the positioning and grasping coordinates of the peach, and obtain and analyze the positioning accuracy.

**Figure 9:**
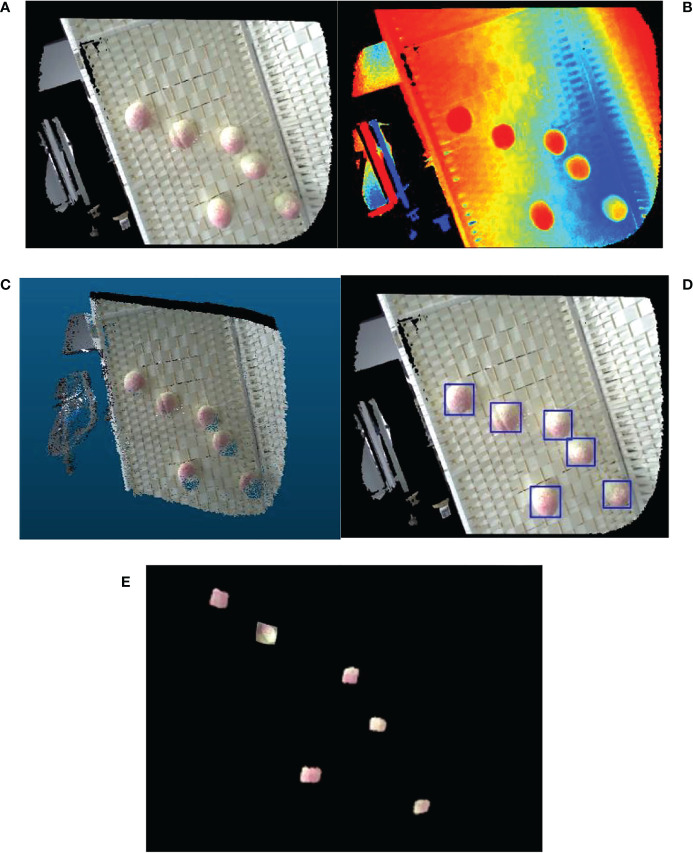
Peaches identification and localization **(A)** Peach color map **(B)** Peach depth map **(C)** Color point cloud image of peach **(D)** Identification map of peach **(E)** Peach point cloud segmentation map.

Comparing the peach positioning result in **Figure 9** with the real value, the positioning accuracy error of peach is obtained. As shown in **Table 5**, it can be seen that the positioning error of peach is basically within 5mm, which can meet the accuracy requirements of peach picking operation.

**Table 5:** The actual and calculated coordinates of the peach and the positioning accuracy error (E).

	peach serial number							
		1	2		3	4	5	6
Real location coordinates of the peach	x	-62.557	-57.891		-44.9571	-38.357	-30.957	-48.156
	y	159.054	162.051		167.874	168.2513	187.2056	177.057
	z	872.957	874.205		879.576	879.981	883.912	875.954
Robot positioning coordinates in this paper	x	-63.8312	-56.201		-45.3171	-40.269	-30.156	-49.8564
	y	159.374	163.997		165.215	170.794	184.457	178.951
	z	869.354	871.731		875.956	876.797	880.591	873.059
E/mm		3.835	3.569		4.5063	4.8327	4.385	3.8548

## 5 Summary

Aiming at the problem of great influence in the outdoor picking environment, this paper proposes a six-axis robot hand-eye calibration method based on TOF depth camera. The hand-eye data is obtained by shooting and extracting the fixed mark points on the calibration board and contact positioning, so as to obtain the hand-eye data. Find the hand-eye calibration matrix. The weighting idea is proposed to optimize the hand-eye parameters, and the experimental platform is used to carry out the hand-eye calibration experiment and the peach picking experiment. The experiments show that the method proposed in this paper has good stability, reliability and good calibration accuracy, which can meet the operation of peach picking require.

## Data availability statement

The raw data supporting the conclusions of this article will be made available by the authors, without undue reservation.

## Author contributions

All persons who meet authorship criteria are listed as authors, and all authors certify that they have participated sufficiently in the work to take public responsibility for the content, including participation in the concept, design, analysis, writing, or revision of the manuscript. All authors contributed to the article and approved the submitted version.
